# GRACE-FO Satellite Data Preprocessing Based on Residual Iterative Correction and Its Application to Gravity Field Inversion

**DOI:** 10.3390/s25113555

**Published:** 2025-06-05

**Authors:** Shuhong Zhao, Lidan Li

**Affiliations:** School of Geological Engineering and Geomatics, Chang’an University, Xi’an 710064, China; shzhao@chd.edu.cn

**Keywords:** satellite gravimetry, data preprocessing, energy balance method, gravity field inversion, spherical harmonic coefficients

## Abstract

To address the limited inversion accuracy caused by low-fidelity data in satellite gravimetry, this study proposes a data preprocessing framework based on iterative residual correction. Utilizing Level-1B observations from the Gravity Recovery and Climate Experiment Follow-On (GRACE-FO) satellite (January 2020), outliers were systematically detected and removed, while data gaps were compensated through spline interpolation. Experimental results demonstrate that the proposed method effectively mitigates data discontinuities and anomalous perturbations, achieving a significant improvement in data quality. Furthermore, a 60-order Earth gravity field model derived via the energy balance approach was validated against contemporaneous models published by the University of Texas Center for Space Research (CSR), German Research Centre for Geosciences (GFZ), and Jet Propulsion Laboratory (JPL). The results reveal a two-order-of-magnitude enhancement in inversion precision, with model accuracy improving from 10^−6^–10^−7^ to 10^−8^–10^−9^. This method provides a robust solution for enhancing the reliability of gravity field recovery in satellite-based geodetic missions.

## 1. Introduction

Satellite gravity observations are pivotal for advancing our understanding of mass redistribution and transport within the Earth system, particularly in monitoring long-term variations in water mass changes across the hydrosphere, oceans, and cryosphere. These observations provide critical constraints on dynamic processes such as ice sheet melt, groundwater depletion, and sea-level rise [1]. Furthermore, the continuous refinement of background geophysical models—essential for isolating subtle gravitational signals from noise—relies heavily on advancements in data computation techniques. The Earth’s gravity field is a fundamental geophysical observable that plays a critical role in revealing terrestrial internal structures, monitoring crustal movements, and analyzing global deformation. To develop high-resolution and high-precision gravity field models, accurate and reliable data are essential [2]. Gravimetric observation data are obtained through measurements by gravity instruments on the ground or in the air and can be categorized into the following types: Terrestrial gravity data: Collected at various ground locations to map the spatial distribution of the gravity field. These data provide high spatial resolution but are significantly influenced by topography and terrain. Airborne gravity data: Acquired using instruments mounted on aircraft, offering large-scale coverage with relatively high spatial resolution. However, the effects of flight altitude and trajectory on data accuracy must be considered. Satellite gravity data: Measured by onboard satellite instruments, enabling global coverage with high precision and detailed information. Consequently, satellite data are predominantly utilized to derive gravity field models. However, due to inherent limitations in these methodologies, their measurement accuracy struggles to meet modern demands [3]. Since the 21st century, advancements in Satellite-to-Satellite Tracking (SST) technology have significantly enhanced gravity field measurement accuracy [4], delivering high-precision observational constraints for global gravity field refinement. The GRACE-FO mission, jointly implemented by the National Aeronautics and Space Administration (NASA) and GFZ, continues the legacy of GRACE by monitoring Earth’s time-variable gravity field and climatic changes. Nevertheless, in-orbit satellites experience atmospheric drag, instrument thermal drifts, and attitude determination residuals during operations, introducing noise, systematic biases, and outliers (termed “anomalous observations”, spikes, and discontinuities) into raw datasets. These anomalies can severely degrade gravity field inversion accuracy. Consequently, robust data preprocessing serves as a critical prerequisite to enhance inversion reliability and mitigate uncertainty. Furthermore, preprocessing provides high-quality input datasets for inversion algorithms and facilitates optimal regularization parameter selection, thereby improving numerical stability and physical consistency in results.

Enhanced numerical algorithms, high-performance computing frameworks, and error mitigation strategies collectively enable the extraction of higher-resolution gravity field solutions, thereby driving progress in quantifying and attributing global mass balance changes with unprecedented accuracy. In recent years, numerous scholars [5,6,7] have conducted in-depth research on satellite data preprocessing. For instance, Kornfeld et al. performed a detailed analysis of data preprocessing techniques for the GRACE-FO mission, exploring how to enhance gravity field recovery accuracy through improved data processing algorithms. Additionally, Goswami et al. investigated ranging signal delay issues in GRACE satellites and proposed a novel delay correction model, significantly reducing systematic errors in the data. Wegener et al. focused on accelerometer data preprocessing for GRACE satellites, developing effective filtering algorithms by analyzing high-frequency noise characteristics. These studies have laid a crucial theoretical foundation and provided technical support for high-quality processing of satellite gravimetry data. Misfeldt et al. advanced multiple methodologies for Laser Ranging Interferometer (LRI) data preprocessing, including phase jump mitigation [8], scale factor estimation [9], and light-time correction [10]. These advancements have substantially improved the precision and reliability of LRI data. The Technical University of Munich (TUM) has developed a time-domain regularized energy method, which addresses the noise sensitivity issue in short-arc data by applying varying constraints to stochastic orbital parameters to establish distinct orbital parameterizations. This approach enhances the robustness of orbital solutions under noisy observational conditions [11]. Zhang Yuanyuan et al. [12] applied the Gaussian filtering method combined with two distinct decorrelation filtering approaches proposed by Swenson and Wahr to process GRACE satellite data. The results demonstrated that this hybrid approach outperformed the combined P4M6 decorrelation filtering and Gaussian filtering method. However, striping artifacts persisted when a smoothing radius of 300 km was applied. Subsequent refinements to the filtering methodology led to the development of a hybrid Gaussian filtering method, which exhibited superior destriping performance compared to the conventional Gaussian smoothing approach. Ma Fuli et al. [13,14] developed a processing chain for gravitational wave high-energy Electromagnetic Counterpart All-sky Monitor satellite data to address low-latency requirements and multi-channel fusion. Zheng Wei et al. [15] utilized the gravity gradient method from GRACE Follow-On satellites to achieve precise inversion of Earth’s gravity field, demonstrating at least a tenfold improvement in accuracy compared to current GRACE satellite solutions. Luo Zhicai et al. [16,17] provided a comprehensive discussion on preprocessing schemes, time-variable gravity field signal corrections, gross error detection, and external calibration methods for GOCE gravity gradient measurements. The JPL [18] developed the Atmosphere and Ocean De-aliasing product (AOD1B), which integrates the ERA5 atmospheric model and FES2014 ocean tide model to mitigate high-frequency mass variation aliasing in gravity field recovery. This study discussed the existing challenges in the inversion of time-variable gravity solutions and explored the feasibility of detecting signals from Earth’s deep interior.

In satellite gravimetry, the inversion accuracy of gravity field models is fundamentally determined by the quality of raw observational data. However, GRACE-FO Level-1B orbital data frequently exhibit significant high-frequency noise and systematic biases in gravity field inversion results due to instrument noise, signal interruptions, and anomalous point disturbances. Partial data gaps in satellite data products substantially impede precise gravity field computation and analysis. To address this limitation, SHUANG et al. developed a non-parametric, data-adaptive Singular Spectrum Analysis (SSA) gap-filling technique, rigorously validated for its dual capability in noise suppression and geophysical signal preservation [19]. In parallel, Hugo systematically evaluated multiple interpolation strategies for reconstructing missing temporal segments in time-variable gravity fields, including least-squares fitting, Principal Component Analysis (PCA), SSA, Multi-Channel SSA (M-SSA), and autoregressive models [20]. These foundational studies established an optimized methodological framework for gap handling, achieving a principled trade-off between computational efficiency and physical consistency with mass flux dynamics. This study proposes a data preprocessing framework based on residual iterative correction, systematically enhancing data quality through outlier removal and data interpolation repair strategies. Experimental validation using GRACE-FO Level-1B orbital data demonstrates the efficacy of this preprocessing approach in improving gravity field inversion quality.

## 2. Materials and Methods

### 2.1. Common Outlier Processing Methods

Outliers are typically addressed through direct removal, which involves eliminating data points exceeding normal ranges along with their neighboring points (defined as the outlier neighborhood). This method requires two critical parameters: the threshold and the neighborhood range.

1. Threshold Selection:

The threshold is determined based on data characteristics, outlier quantity, and distribution. Statistical properties (e.g., mean, median, standard deviation) are commonly used to derive appropriate thresholds. For datasets with distinct boundaries, thresholds can be directly specified according to application requirements.

**Figure 1:**
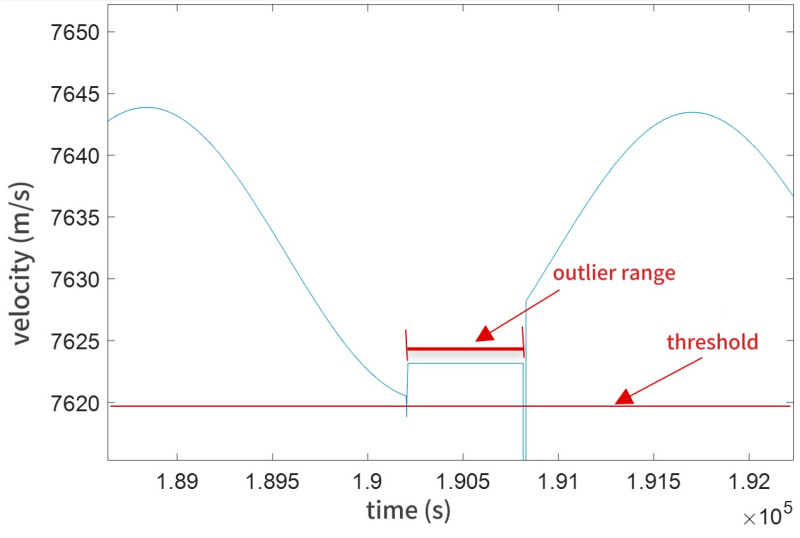
Threshold and outlier range.

2. Neighborhood Range Optimization:

The spatial characteristics of outliers dictate the neighborhood range: Clustered outliers: A smaller neighborhood is sufficient to remove proximate anomalous points. Scattered outliers: A larger neighborhood ensures comprehensive outlier elimination. The principle is illustrated in Figure 1. The process iteratively adjusts these parameters to balance outlier removal efficacy and data integrity preservation.

**Figure 2:**
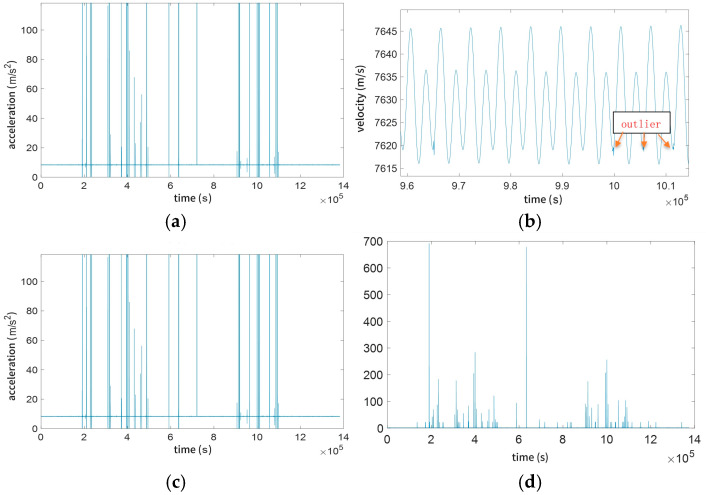
Threshold and outlier range; (**a**) velocity magnitude; (**b**) filtered velocity magnitude; (**c**) acceleration magnitude; (**d**) differential processing of time series.

The primary raw data requiring processing include temporal data, velocity data, and positional data, with subsequent considerations for acceleration data and post-filtering datasets. Each data type necessitates distinct thresholds and neighborhood ranges for outlier elimination, as illustrated in Figure 2.

Some outliers exist within the normal value range of the data, where their values are close to neighboring valid data, making threshold determination challenging (Figure 2b). By differentiating the time series after outlier removal, the processed data exhibit poor continuity, as shown in Figure 2d. In gravity field inversion using the energy balance approach, high data continuity is critical, and shorter continuous data segments result in larger errors in constant terms. Therefore, interpolation is required for the data after outlier removal. Additionally, differentiation of the time series reveals significant gaps in the processed data. Given the limitations of direct outlier removal, conventional interpolation methods are inadequate. The methodology adopted in this study involves iterative fitting to identify and remove outliers. For data gaps, fitted results are used as replacements. To minimize the impact of fitted data on inversion results, reduced weights are assigned to these fitted values during inversion.

### 2.2. The Process of Data Preprocessing

**Figure 3:**
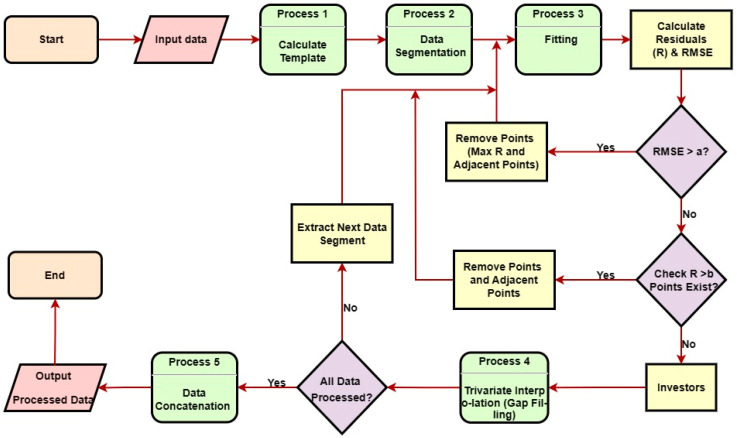
Data preprocessing flowchart.

The data preprocessing employs iterative fitting procedures structured into five stages. First, the input data are partitioned into segments. During each iteration, residuals (R) between the data points and fitted results are quantified alongside the root mean square error (RMSE). Based on the magnitude of RMSE, two scenarios are categorized for outlier removal: High-Impact Segments (RMSE > a): The data point with the maximum residual and adjacent points within a specified window are discarded to mitigate significant fitting distortions; low-Impact Segments (RMSE < a): A residual threshold b is defined. Points exceeding R > b are filtered out; otherwise, multivariate spline interpolation is applied for gap compensation. The iterative process continues until predefined accuracy criteria are met. Processed segments are then concatenated and aggregated for output (Figure 3). This framework balances fitting accuracy and computational efficiency while ensuring robustness against data anomalies.

**Figure 4:**
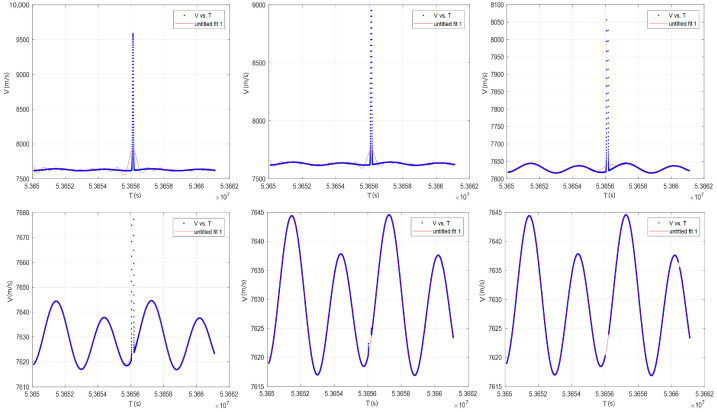
Effectiveness of iterative fitting in removing data outliers.

During outlier detection and removal, the processing targets the magnitude of the input data. After outlier elimination, three-component fitting-based interpolation is applied. The data fitting method employed in this study is Fourier series fitting, where the data length is closely related to the order of the fitting. For longer data sequences, achieving equivalent fitting accuracy necessitates higher-order fitting, which incurs greater computational load. The processed data span 10,000 s, with a fitting order of 9. The iterative fitting approach for outlier removal achieves robust results, and the processed data exhibit no discontinuities, as demonstrated in Figure 4.

### 2.3. Basic Principle of Energy Method Inversion

The theoretical foundation of the energy balance inversion method is rooted in the many-body system energy conservation principle within classical celestial mechanics. As a satellite traverses the Earth’s gravitational field, its orbital dynamics are governed by gravitational forces, centrifugal effects, Coriolis accelerations, and non-conservative perturbations. To formulate a precise dynamical model, Lagrangian mechanics and Hamiltonian formalism are employed to characterize the satellite’s motion and derive its energy conservation equation. Orbital data fidelity plays a pivotal role in gravity field recovery; thus, to quantify the impact of data quality on inversion accuracy, the energy method is applied to reconstruct the Earth’s gravity field from single-satellite observations. The observation equations in the Earth-fixed reference frame are given by the Equation (1) [21].

(1) $$T=\frac{1}{2}{\dot{r}}^{e}\cdot {\dot{r}}^{e}-\frac{1}{2}(\omega_{\mathrm{ie}}^{e}\times r^{e})\cdot (\omega_{\mathrm{ie}}^{e}\times r^{e})-\int_{t_{0}}^{t}a_{1}^{e}\cdot {\dot{r}}^{e}d\tau -\int_{t_{0}}^{t}a_{2}^{e}\cdot {\dot{r}}^{e}d\tau -U_{0}-E^{const}$$

where $E^{const}$ is the specific mechanical energy constant, $U_{0}$ denotes the Earth’s gravitational potential, $r^{e}$ and ${\dot{r}}^{e}$ represent the position and velocity vectors of the satellite, respectively, $\omega_{\mathrm{ie}}^{e}$ is the Earth’s angular velocity vector, $a_{1}^{e}$ denotes the acceleration induced by non-conservative forces acting on the satellite (such as atmospheric drag, solar radiation pressure, earth radiation pressure, etc.), while $a_{2}^{e}$ represents the acceleration arising from conservative forces other than Earth’s gravitation (e.g., third-body gravitational perturbations from the Moon and Sun).

The gravitational potential V at any point on Earth is typically expressed through a finite-order spherical harmonic expansion:

(2) $$V(r,\theta ,\lambda )=\frac{GM}{r}{\left\{1+\sum_{n=2}^{\infty }\sum_{m=0}^{n_{\max}}[\frac{R_{e}}{r}\right]}^{n}({\bar{C}}_{\mathrm{nm}}\cos m\lambda +{\bar{S}}_{\mathrm{nm}}\sin m\lambda ){\bar{P}}_{\mathrm{nm}}(\cos\theta \left.)\right\}$$

where G is the gravitational constant (6.67430 × 10^−11^ m^3^·kg^−1^·s^−2^), M is the Earth’s mass, and $R_{e}$ is the mean radius of the Earth (6.378137 × 10^6^ m); r, θ and λ are geocentric spherical coordinates (radius, colatitude, and longitude, respectively); ${\bar{C}}_{\mathrm{nm}}$ and ${\bar{S}}_{\mathrm{nm}}$ are fully normalized spherical harmonic coefficients; and ${\bar{P}}_{\mathrm{nm}}$ is the fully normalized associated Legendre functions.

To facilitate numerical computations, Equation (2) is transformed into the Cartesian coordinate system:

(3) $$V(X,Y,Z)=\frac{GM}{R_{e}}\sum_{n=0}^{\infty }\sum_{m=0}^{n_{\max}}(E_{\mathrm{nm}}{\bar{C}}_{\mathrm{nm}}+F_{\mathrm{nm}}{\bar{S}}_{\mathrm{nm}})$$

where $\left(X,Y,Z\right)$ are the three components of the position vector in the Cartesian coordinate system; V is the Earth’s gravitational potential, and $E_{\mathrm{nm}}$ and $F_{\mathrm{nm}}$ are functions analogous to the associated Legendre polynomials, which can be derived via the following recurrence relation [4]:

(4) $$\begin{array}{l} E_{0,0}=\frac{R_{e}}{r},F_{0,0}=\frac{R_{e}}{r}\\ E_{n,n}=(2n-1)R_{e}[\frac{x}{r^{2}}E_{n-1,n-1}-\frac{y}{r^{2}}F_{n-1,n-1}]\\ F_{n,n}=(2n-1)R_{e}[\frac{x}{r^{2}}E_{n-1,n-1}+\frac{y}{r^{2}}F_{n-1,n-1}]\\ E_{n,m}=\frac{R_{e}}{n-m}(2n-1)\frac{z}{r^{2}}E_{n-1,m}\;n=m+1\\ F_{n,m}=\frac{R_{e}}{n-m}(2n-1)\frac{z}{r^{2}}F_{n-1,m}\;n=m+1\\ E_{n,m}=\frac{1}{n-m}[(2n-1)R_{e}\frac{z}{r^{2}}E_{n-1,m}-\frac{\left(n+m-1\right)}{r^{2}}R_{e}^{2}E_{n-2,m}]\\ F_{n,m}=\frac{1}{n-m}[(2n-1)R_{e}\frac{z}{r^{2}}F_{n-1,m}-\frac{\left(n+m-1\right)}{r^{2}}R_{e}^{2}F_{n-2,m}] \end{array}$$

where $r=\sqrt{x^{2}+y^{2}+z^{2}}$.

By neglecting the zeroth-order term in Equation (3), i.e., the normal gravitational potential, and since the first-order term of the spherical harmonic coefficient of the Earth’s gravitational field is zero, the perturbation potential T of the Earth’s gravitational field can be expressed as.

(5) $$T(X,Y,Z)=\frac{GM}{R_{e}}\sum_{n=2}^{\infty }\sum_{m=0}^{n_{\max}}(E_{\mathrm{nm}}{\bar{C}}_{\mathrm{nm}}+F_{\mathrm{nm}}{\bar{S}}_{\mathrm{nm}})$$

From Equation (1), it can be seen that utilizing the energy method will involve multiple numerical integrations, and the presence of anomalous data points will severely degrade the accuracy of the computed disturbing potential T. Therefore, eliminating the influence of such outliers is critical for ensuring the fidelity of the inversion results.

To further quantify model consistency, the order variances of the spherical harmonic coefficients of the two models with the spherical harmonic coefficients of the GFZ model are calculated, and the results are shown in Figure 9. Where the order variances of the model are calculated as follows:

(6) $$RMS_{n}=\sqrt{\frac{\sum_{m=0}^{n}{\left(C_{\mathrm{nm}}-C_{\mathrm{nm}}^{l}\right)}^{2}+\sum_{m=1}^{n}{\left(S_{\mathrm{nm}}-S_{\mathrm{nm}}^{l}\right)}^{2}}{2n+1}}$$

where $C_{\mathrm{nm}},S_{\mathrm{nm}}$ denote the coefficients from our inversion, and $C_{\mathrm{nm}}^{l},S_{\mathrm{nm}}^{l}$ represent the GFZ-published coefficients.

## 3. Experiments and Analysis

### 3.1. GRACE-FO Satellite Data Experiments

**Figure 5:**
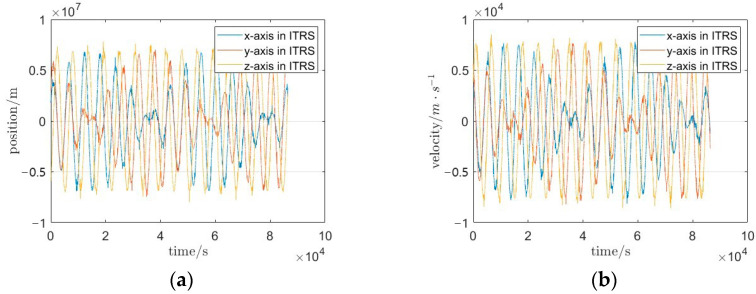
Time series of simulated raw satellite orbital data (1-day interval); (**a**) time series of triaxial position components with artificial perturbations and gaps; (**b**) time series of triaxial velocity components with artificial perturbations and gaps.

Due to restricted access to GRACE-FO Level-0 raw telemetry data, the Level-1B orbital products from January 2020 were selected as the raw dataset, and to emulate mission-representative data anomalies, artificial gaps and perturbations were introduced into the original dataset. The resulting perturbed triaxial position and velocity time series are illustrated in Figure 5.

**Figure 6:**
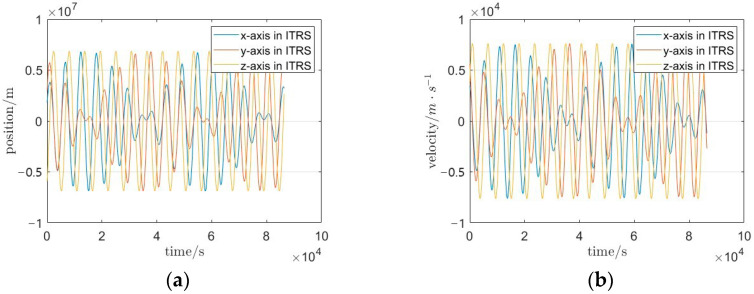
Time series of preprocessed satellite orbit data (1-day interval); (**a**) post-preprocessing time series of triaxial position components; (**b**) post-preprocessing time series of triaxial velocity components.

**Figure 7:**
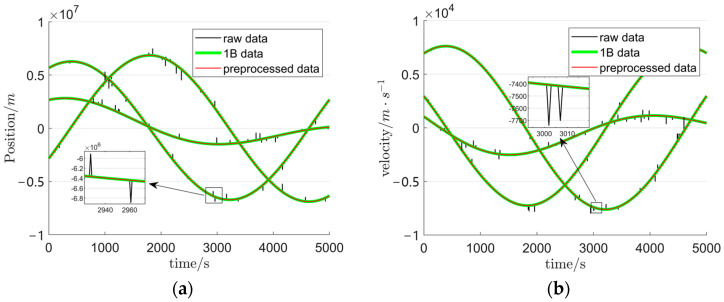
Comparison of triaxial position and velocity components; (**a**) triaxial position component comparison across three datasets; (**b**) triaxial velocity component comparison across three datasets.

The velocity and position data in Figure 5 exhibit a high density of outliers and poor temporal continuity. To address these anomalies, the dataset undergoes preprocessing, with the refined results depicted in Figure 6. For a quantitative comparison of data continuity improvements, 5000-s segments of the simulated data, Level-1B observations, and preprocessed data are extracted. The comparative analysis of their temporal coherence is presented in Figure 7.

**Figure 8:**
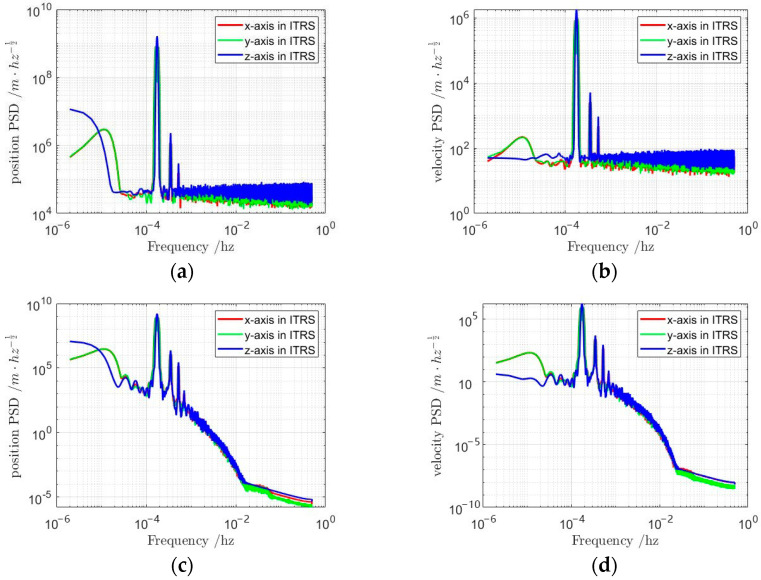
Power spectral density (PSD) of satellite position and velocity in the Earth-Fixed Frame; (**a**) PSD of raw satellite position data; (**b**) PSD of raw satellite velocity data; (**c**) PSD of preprocessed satellite position data; (**d**) PSD of preprocessed satellite velocity data.

A comparative analysis of the three curves in Figure 7 reveals that the preprocessing successfully eliminates the artificially injected perturbations, fills data gaps, and achieves strong consistency between the processed and source datasets. These results demonstrate the efficacy of the proposed preprocessing method in enhancing data quality. Furthermore, power spectral density analyses of preprocessed versus raw velocity and position data are presented in Figure 8. In Figure 8a,b, the diminished power in both low- and high-frequency bands of the raw position/velocity PSD spectra reflects elevated uncertainty and noise levels, indicative of inherent instability in the original dataset. Conversely, the suppressed high-frequency power in Figure 8c,d demonstrates significant mitigation of random orbital errors post-preprocessing, confirming improved orbital stability. This spectral evidence corroborates the successful removal of perturbations and recovery of data continuity.

### 3.2. Inversion Results Based on the Energy Method

The energy balance approach [22] and short-arc integration method [23,24] represent two widely adopted computational methodologies for gravity field model inversion. The energy balance approach computes the disturbing potential by formulating linearized equations derived from satellite velocity and position vectors, thereby establishing connections with gravity field coefficients. This method demonstrates strong parallel processing potential when handling massive observational datasets, making it suitable for large-scale parallel computing. However, it requires numerical differentiation of orbital data, where velocity accuracy directly governs inversion fidelity. In contrast, the short-arc integration method employs linearized equations that circumvent the need for initial gravity field models or iterative computations while avoiding errors introduced by numerical differentiation. Nevertheless, this approach exhibits inherent numerical complexity, where lower-order interpolation at arc segment endpoints degrades integration precision, and extended arc lengths may compromise computational stability.

**Figure 9:**
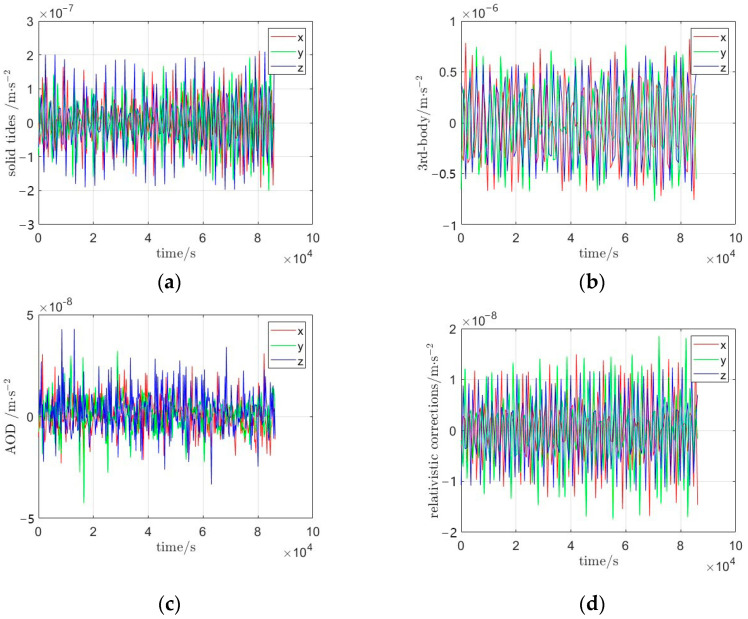
Conservative perturbation contributions in gravity field inversion; (**a**) solid earth/ocean tides; (**b**) third-body gravitational effects (sun/moon); (**c**) atmospheric/oceanic non-tidal variations; (**d**) relativistic corrections.

**Figure 10:**
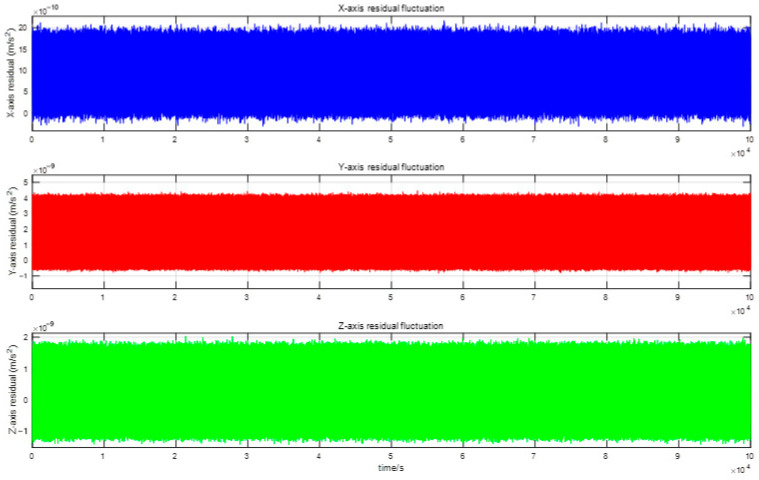
Accelerometer residual fluctuations.

Over time, both the Earth’s interior and surface undergo dynamic changes driven primarily by tidal forces and mass transport processes within and on the Earth. These phenomena, collectively termed time-variable effects, introduce disturbances that must be accounted for to recover high-precision, high-resolution gravity field models. The disturbing potential is derived from the observation equation (Equation (1)). The perturbation models considered in this study include solid Earth and ocean tides, third-body gravitational effects (Sun and Moon), relativistic corrections, and atmospheric/oceanic non-tidal variations [25,26], as illustrated in Figure 9. The corresponding model parameters are summarized in Table 1. Furthermore, direct computation of non-conservative forces from accelerometer data is inherently limited by scale factor errors and systematic biases, necessitating rigorous recalibration of accelerometer measurements during gravity field inversion. And the accelerometer residual fluctuations are shown in Figure 10.

**Figure 11:**
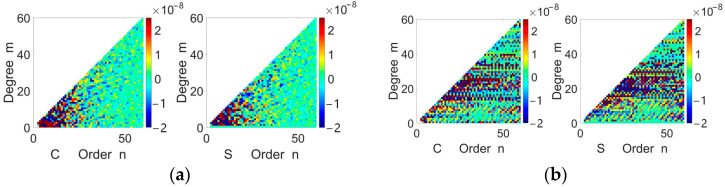
Spherical harmonic coefficients; (**a**) spherical harmonic coefficients derived from preprocessed data; (**b**) spherical harmonic coefficients derived from raw data.

After removing the non-spherical gravitational perturbations computed using the a priori EGM2008 model and the non-conservative force perturbations measured by onboard accelerometers, the disturbing potential T and corresponding satellite orbital positions are substituted into Equation (5). This yields a linear system of equations with the spherical harmonic coefficients of the Earth’s gravity field as variables. The system is solved via first-order Tikhonov regularization within a least-squares framework [27] (to ensure the generalizability of the method, regularization processing was applied), resulting in a set of 60-order spherical harmonic coefficients (excluding order-0 and order-1 terms) as illustrated in Figure 11a.

Figure 11a reveals distinct statistical characteristics of the spherical harmonic coefficients across different spectral bands: Low-order coefficients (order ≤ 10) predominantly cluster near the extremes of the distribution interval and exhibit larger magnitudes. High-order coefficients (order > 10) are concentrated toward the central region of the interval, with magnitudes decaying progressively as the order increases.

To evaluate the impact of data preprocessing, Figure 11b displays the coefficients obtained from the inversion of raw, unprocessed data. A striking contrast emerges: compared to the structured distribution in Figure 11a, the coefficients in Figure 11b show pronounced clustering near the interval boundaries across all orders, indicating amplified high-frequency noise and systematic biases inherent to untreated observations.

The global 1° × 1° grid gravity anomalies are calculated with the model obtained from the inversion of the numbers before and after preprocessing and analyzed in comparison with the gravity anomalies calculated by the 60-order gravity field model of the same period published by CSR, JPL, and GFZ, and their difference results are analyzed statistically (Table 2). The gravity anomalies calculated by 60-order gravity field models published by CSR, JPL, and GFZ for the same period are analyzed in comparison with each other, and the results of their differences are statistically analyzed (Table 2). The tabulated results demonstrate that the preprocessed model exhibits significantly reduced gravity anomaly discrepancies (Δg) relative to all reference models, confirming its enhanced accuracy.

**Figure 12:**
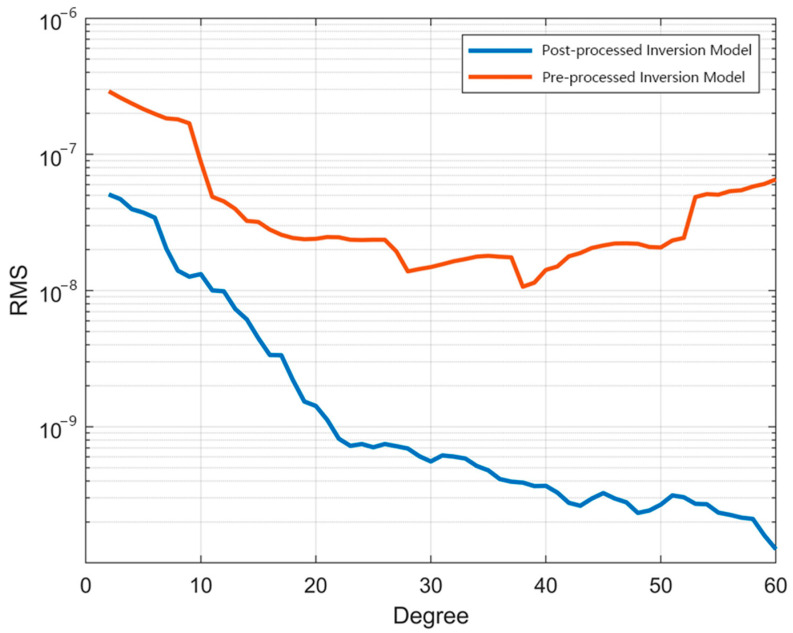
Order variances between the two inverted models and the GFZ reference model.

**Figure 13:**
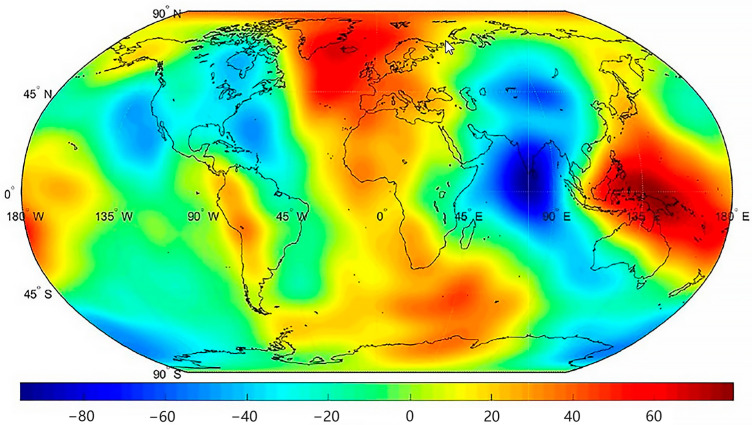
Geoid height anomalies (m).

**Figure 14:**
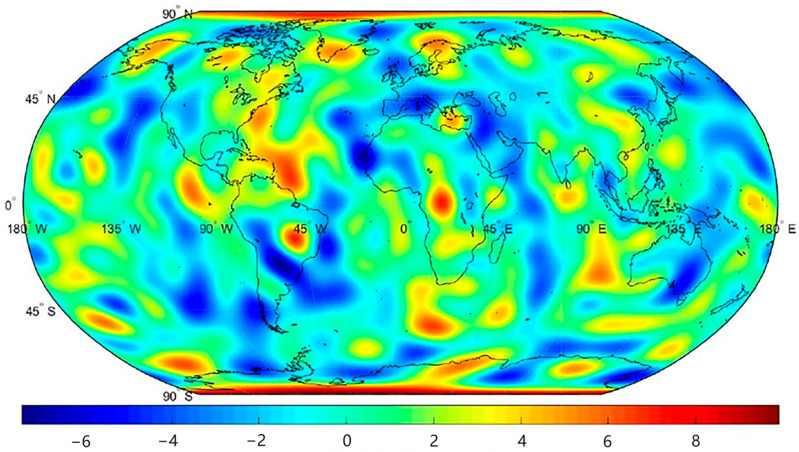
Discrepancy from GFZ model (m).

As shown in Figure 12, the unprocessed-data model shows substantial deviations across both low- and high-order terms (order variance: 10^−6^–10^−7^), rendering it physically implausible for practical applications. In contrast, the preprocessed-data model achieves a remarkable magnitude reduction, with order variances stabilized at 10^−8^–10^−9^, demonstrating enhanced physical consistency with the GFZ benchmark. Finally, geoid anomaly maps were generated from the preprocessed data, and equivalent water height (EWH) maps of residuals between the preprocessed data and GFZ data were plotted to evaluate the effectiveness of the preprocessing framework (Figure 13 and Figure 14). These results conclusively validate that our preprocessing framework effectively suppresses outliers, compensates for data gaps, and improves gravitational field recovery fidelity.

## 4. Conclusions

The preprocessing framework proposed in this study demonstrates robust capability in detecting data gaps and outliers within satellite datasets. By systematically filtering anomalies and compensating gaps through spline interpolation, this method ensures temporal continuity and significantly enhances data quality, thereby establishing a reliable foundation for gravity field inversion. To validate the framework’s efficacy, two 60-order gravity field models were generated using GRACE-FO January 2020 data: Raw Data Model: Simulates low-quality observations by injecting artificial perturbations (e.g., Gaussian noise, periodic spikes) and data gaps (5% temporal discontinuities). Preprocessed Data Model: Applies the proposed method to mitigate anomalies and recover continuity. Both models were inverted via the energy balance approach and compared against contemporaneous 60-order reference models from CSR, JPL, and GFZ. The analysis reveals: The raw-data-derived model exhibits poor accuracy (10^−6^–10^−7^ order of magnitude), rendering it inadequate for geophysical applications. In contrast, the preprocessed model achieves precision improvements of two orders of magnitude (10^−8^–10^−9^), aligning closely with the reference models. These results conclusively demonstrate that the preprocessing method effectively suppresses noise artifacts, preserves data integrity, and enables high-fidelity gravity field recovery. In addition to the data presented in this study, the proposed approach was validated using data from the Taiji satellites, achieving satisfactory results. This demonstrates that preprocessing based on residual iterative data can effectively mitigate the impact of outliers on gravity field inversion.

## Figures and Tables

**Table 1:** Explanation of Inversion Parameters for GRACE-FO Satellite Data in January 2020.

Parameter Name	Parameter Details
Inversion Order	60 Order
Background Models for Inversion	EGM2008 Model (6–100 Order)
	Third-body perturbations (Sun, Moon; JPL DE421 ephemeris)
	Solid Earth Tides (first 4 orders; IERS 2010 conventions)
	AOD1B product (RL06)
	Relativistic Effects (IERS 2010 conventions)
Data Sampling Interval	1 s
Data Duration	30 days
Data Period	January 2020

**Table 2:** The statistical results of the gravity anomaly difference (mGal) between the models and each model before and after pretreatment.

Gravity Field Model	Models Obtained Before Preprocessing				Model Obtained After Preprocessing			
	Min	Max	Mean	Std	Min	Max	Mean (10^−2^)	Std
CSR	−1280.728	2140.733	184.786	11.784	−389.5214	216.8015	5.22	6.1981
JPL	−1280.884	2140.387	184.894	11.856	−389.3761	216.5672	5.58	6.1957
GFZ	−1280.435	2140.956	184.265	11.257	−389.9654	216.3126	5.37	6.1948

## Data Availability

Data are contained within the article.
